# Hypertensive Response to Exercise in Normotensive Men and Women with Abdominal Obesity: Association with Subclinical Adverse Cardiac Remodeling

**DOI:** 10.3390/jcm14010016

**Published:** 2024-12-24

**Authors:** Amélie Paquin, Marie-Anne Mathieu, Chloé Prémont, Iris Gigleux, Anne-Sophie Neyron, Maggie Lê-Brassard, Mickaël Martin, Audrey Auclair, Myriam Pettigrew, Robert Ross, Patrick Couture, Jean-Pierre Després, Paul Poirier, Benoît Lamarche, Marie-Eve Piché

**Affiliations:** 1 Institut Universitaire de Cardiologie et de Pneumologie de Québec, Université Laval, Québec, QC G1V 4G5, Canada; amelie.paquin@criucpq.ulaval.ca (A.P.); marie-anne.mathieu.1@ulaval.ca (M.-A.M.); chloe.premont@criucpq.ulaval.ca (C.P.); mickael.martin@criucpq.ulaval.ca (M.M.); audrey.auclair@criucpq.ulaval.ca (A.A.); myriam.pettigrew@criucpq.ulaval.ca (M.P.); jean-pierre.despres@criucpq.ulaval.ca (J.-P.D.); paul.poirier@criucpq.ulaval.ca (P.P.); 2 Faculty of Medicine, Université Laval, Québec, QC G1V 0A6, Canada; patrick.couture@fmed.ulaval.ca; 3 Centre Nutrition, Santé et Société (NUTRISS), Institut sur la Nutrition et les Aliments Fonctionnels (INAF), Université Laval, Québec, QC G1V 0A6, Canada; iris.gigleux@fsaa.ulaval.ca (I.G.); anne-sophie.neyron@fsaa.ulaval.ca (A.-S.N.); maggie.le-brassard.1@ulaval.ca (M.L.-B.); benoit.lamarche@fsaa.ulaval.ca (B.L.); 4 School of Medicine, Division of Endocrinology and Metabolism, Queen’s University, Kingston, ON K7L 3N6, Canada; rossr@queensu.ca; 5 VITAM—Centre de Recherche en Santé Durable, Université Laval, Québec, QC G1V 0A6, Canada; 6 Faculty of Pharmacy, Université Laval, Québec, QC G1V 0A6, Canada

**Keywords:** hypertensive response to exercise, cardiorespiratory exercise testing, echocardiography, abdominal obesity

## Abstract

**Background/Objectives:** Hypertensive response to exercise (HRE) is an established risk factor for cardiovascular events. HRE is prevalent among people with excess adiposity. Both obesity and HRE have been individually associated with adverse cardiac remodeling. We hypothesized that HRE would be associated with adverse measures of cardiac structure and function in a subgroup of individuals with abdominal obesity. **Methods:** In a subgroup of 158 participants with elevated waist circumference (women: ≥80 cm, men: ≥94 cm) and resting blood pressure (BP) < 140/90 mm Hg, we evaluated maximal exercise BP and peak oxygen consumption (VO_2peak_) during cardiorespiratory exercise testing. HRE was defined as maximal exercise BP ≥ 90th percentile per sex and age. Cardiac structure and function on echocardiography were compared between HRE and no HRE (NHRE) groups for each sex. Multivariate linear regression was used to evaluate associations between maximal systolic BP (SBP) and echocardiographic variables, adjusting for age, body mass index, resting SBP, and VO_2peak_. **Results:** Participants (75% women) were aged 53 ± 11 years old. Women with HRE had higher resting SBP and subclinical cardiac remodeling abnormalities (increased left ventricular [LV] wall thickness, relative wall thickness, and mass) than NHRE women (*p* < 0.05). Men with HRE had higher relative wall thickness than NHRE men (*p =* 0.042). There was no difference in cardiac function between HRE groups (*p* > 0.05). After adjustment, maximal SBP remained associated with adverse cardiac remodeling parameters for each sex (*p* < 0.05). **Conclusions:** Among individuals with abdominal obesity and resting BP < 140/90 mm Hg, HRE was associated with adverse cardiac remodeling. HRE represents a potential screening tool and preventive target to detect those at higher risk of preclinical cardiac changes.

## 1. Introduction

It is a well-established phenomenon that systolic blood pressure (SBP) increases during exercise in response to the higher oxygen demands placed on muscles [1,2]. In contrast, diastolic blood pressure (DBP) typically remains stable or even decreases due to peripheral vasodilatation [1,2]. Hypertensive response to exercise (HRE) is often defined by a maximal SBP and/or a maximal DBP exceeding the 90th to 95th percentile for a given sex and age category [2,3,4,5,6,7,8]. The two definitions of HRE most commonly utilized are as follows: (1) SBP ≥ 210 mm Hg (men), SBP ≥ 190 mm Hg (women) and/or DBP ≥ 110 mm Hg [2,3,4,5,6,7], and (2) the value corresponding to the 90th percentile maximal blood pressure (BP) according to sex and age [8]. HRE is associated with an increased risk of developing chronic arterial hypertension (HTN) in healthy normotensive men and women [9,10]. Furthermore, an HRE has been linked to several other cardiovascular risk factors, such as insulin resistance, dyslipidemia and the metabolic syndrome, [11,12,13,14] and to an elevated risk of cardiovascular events in diverse populations [15,16,17].

Excess adiposity and obesity have been associated with an increased risk of HRE [7] even in the absence of clinical HTN. More specifically, abdominal obesity is associated with high sympathetic nervous activity [18,19], endothelial dysfunction, abnormalities of the renin angiotensin aldosterone system [20], and a chronic inflammatory state [21]. All those mechanisms are linked with BP and could contribute to HRE in individuals with abdominal obesity.

Similarly to HTN, both obesity and HRE have been linked to the development of adverse cardiac structural and functional changes [3,22,23,24,25,26,27,28,29]. These changes include increased left ventricular (LV) posterior wall thickness, higher LV mass [3,28], and higher prevalence of LV hypertrophy [27]. In response to chronic HTN, the pattern of cardiac remodeling appears different between sexes, with a tendency to develop more eccentric LV hypertrophy in men compared to women who tend to develop more concentric LV hypertrophy [30]. However, despite these differences between men and women, there are currently no sex-specific nor sex-stratified data regarding the association of HRE with parameters of adverse cardiac remodeling and cardiac function. The objective of this study was to compare the metabolic profile, cardiopulmonary exercise testing variables and cardiac structure and function of men and women with abdominal obesity and resting BP < 140/90 mm Hg, according to the presence or absence of HRE. The secondary objective was to explore the relationship between maximal exercise SBP and cardiac structure and function specific to sex. According to the current literature, our hypothesis is that HRE will be associated with more altered measurements of cardiac structure and function compared to those without HRE.

Practitioner Points:
  - A significant proportion of hypertensive response to exercise can be found among individuals with abdominal obesity and without prior clinical diagnosis of hypertension.
  - Hypertensive response to exercise is associated with more adverse remodeling of the left ventricle.
  - Understanding that early identification of left ventricular remodeling can identify patients at higher risk of future cardiovascular disease, hypertensive response to exercise could help target patients who would benefit the most from preventive strategies among those affected by abdominal obesity.

## 2. Material and Methods

### 2.1. Participants and Study Design

A secondary cross-sectional analysis was conducted on the baseline characteristics and test results of a randomized controlled trial (clinical trial registration number: NCT03731013). A total of 200 participants with abdominal obesity (waist circumference ≥80 cm in women, ≥94 cm in men according to the definition of the metabolic syndrome proposed by the International Diabetes Federation) [31] and with fasting serum triglycerides (TG) ≥1.5 mmol/L (threshold associated with smaller, denser and more atherogenic low-density lipoprotein [LDL] particles) [32] were enrolled. Individuals with a prior history of cardiovascular disease, diabetes, use of anti-diabetic drugs or medication affecting the metabolic profile in the past six months, weight loss of ≥5 kg in the previous three months, ≥100 min of regular moderate-intensity physical activity, or a Mediterranean Diet score of at least 20 [33] were excluded from the study. For the present secondary ad hoc analysis, participants with a resting BP ≥ 140/90 mm Hg or those using anti-hypertensive medication (*n =* 33), and those without available echocardiographic data (*n =* 9) were additionally excluded. The protocol was approved by the Research Ethics Committee of the Institut Universitaire de Cardiologie et de Pneumologie de Québec—Université Laval (2019-3061, 21628). All participants provided written informed consent. This article is a revised and expanded version of a paper entitled “Association of exercise blood pressure response with subclinical abnormalities of cardiac remodeling and function in middle-aged women,” which was presented at the European Society of Cardiology Preventive Cardiology Scientific Meeting, Athens (Greece), in 2024 [34].

### 2.2. Anthropometric Measurements and Resting Measures

Information regarding the participants’ prior medical history, medication use, and lifestyle habits was collected at the baseline visit. Anthropometric measurements were obtained using a standardized protocol [35]. Height was measured with a stadiometer. Body weight was obtained with a digital scale (Tanita BWB-800, Tanita Corporation, Tokyo, Japan). Three measurements of waist and hip circumferences were taken using a measuring tape [36], and the mean was calculated to determine the waist-to-hip ratio. Body mass index (BMI) was calculated as body weight (kg) divided by squared height (m^2^). Following a 10 min rest period, resting BP and heart rate were measured three times, with five minutes between each measurement, and averaged using an automated BP monitor (Omron HEM-907XL) at the Institut sur la Nutrition et les Aliments Fonctionnels (INAF).

### 2.3. Biochemical Analysis

Blood samples were drawn after 12 h fasting and included fasting plasma lipid-lipoprotein profile (high density lipoprotein cholesterol [HDL-C], LDL-cholesterol, TG), apolipoprotein B (apoB), glucose, insulin, and glycated hemoglobin (HbA1c). Insulin resistance was calculated with the homeostasis model assessment method (HOMA-IR) [37]. Fasting plasma C-reactive protein (CRP) was measured using the Behring Latex-Enhanced (highly sensitive) CRP (Hs-CRP) assay on the Behring Nephelometer BN-100 (Behring Diagnostic, Westwood, MA, USA) and the calibrators (N Rheumatology Standards SL) provided by the manufacturer [38].

### 2.4. Cardiorespiratory Exercise Testing

Cardiorespiratory fitness was assessed during a maximal exercise treadmill cardiorespiratory exercise testing with gas exchange analysis, using the modified Bruce protocol [39] at baseline visit. The test was administered by a trained clinical exercise physiologist, according to the American College of Sports Medicine, and supervised by a cardiologist. Breath-by-breath expired gases were analyzed using a metabolic cart (Medgraphics, Ultima CariO_2_, MGC Diagnosis, Saint Paul, MN, USA) to determine the absolute (mL O_2_/min) and relative (indexed to body weight, mL O_2_/kg/min) maximal oxygen consumption measured (VO_2peak_). Metabolic equivalents were calculated as the relative VO_2peak_ divided by 3.5 mL O_2_/kg/min. Heart rate was monitored with a 12-lead electrocardiogram (Cardioperfect Welch-Allyn, Skaneateles Falls, NY, USA) throughout the test. BP was measured in the pretest sitting position every 3 min before the end of each exercise stage and during recovery using an automated sphygmomanometer with a headphone circuit option (Model 412, Quinton Instrument, Bothell, WA, USA) [40]. Maximal BP corresponds to the maximal BP obtained during exercise or in the first minute of recovery. HRE was defined as a maximal SBP ≥ 90th percentile per sex and age category as previously reported [8]. Maximal double product corresponds to the product of maximal SBP and maximal heart rate [39,41]. Delta SBP corresponds to the difference between maximal and resting SBP. The workload-indexed BP response corresponds to the ratio between the delta SBP and delta metabolic equivalent (METs) of task (maximal METs-resting METs).

### 2.5. Transthoracic Echocardiography

Transthoracic echocardiography was performed according to the recommendations from the American Society of Echocardiography [42] with a commercially available ultrasound system (Vivid 9; GE Healthcare, Ultrasound AS, Horten, Norway) at baseline study visit. LV dimensions were measured in the parasternal long-axis view. LV mass was calculated using the formula proposed by Devereux and Reichek and indexed to body surface area [43]. Concentric remodeling refers to a normal indexed LV mass (≤95 g/m^2^ for women and ≤115 g/m^2^ for men) but with an abnormal relative wall thickness (>0.42) [42].

LV ejection fraction was quantified using the modified biplane Simpson method [42]. The subclinical cardiac function (global longitudinal myocardial strain [GLS]) was measured using speckle tracking echocardiography [42]. LV GLS was calculated by averaging the value of all LV myocardial segments from the apical 4-, 3-, and 2- chamber views. GLS measurements are expressed in their absolute value (|%|) [42]. Diastolic function was evaluated by measuring the mitral annular tissue Doppler velocity (lateral and septal e’), mitral inflow E and A velocities, the E/e’ ratio, peak tricuspid regurgitation velocity, and left atrial volume (modified biplane formula, indexed for body surface area) [44].

### 2.6. Statistical Analysis

Continuous variables are presented by mean ± standard deviation. Normality tests were used to verify the distribution of data (Shapiro-Wilk, Kolmogorov-Smimov). Categorial variables are presented by frequencies and percentages. Participants were stratified by sex and compared according to the presence or absence of HRE (HRE vs. NHRE). The independent sample Student’s *t* test was used to compare participant characteristics and echocardiography results between individuals with and without HRE, stratified by sex. A Pearson correlation coefficient was calculated to assess the correlation between maximal SBP and cardiac structure and function. Multivariate linear regression was utilized to evaluate the association between maximal SBP and cardiac structure and function, adjusting for age, BMI, resting SBP, and VO_2peak_. Additionally, an interaction term was tested between maximal SBP and sex to assess for the potential modulating effect of sex on the association between maximal SBP and echocardiographic variables, when significant sex-specific associations were found. A two-sided *p*-value <0.05 was considered as statistically significant. Analyses were conducted using the SPSS statistical software (version 29).

## 3. Results

### 3.1. Participants Clinical Characteristics

A total of 158 participants (118 women, 40 men) aged 54 ± 11 years old, with abdominal obesity (waist circumference of 99.5 ± 9.8 cm for women, and 107.9 ± 7.2 cm for men) and without prior diagnosis of clinical HTN (mean resting SBP 116 ± 10 mm Hg, mean resting DBP: 72 ± 7 mm Hg), were included in this study (Table 1 and Table 2). Women had lower maximal SBP (191 ± 24 mm Hg for women vs. 202 ± 25 mm Hg for men; *p =* 0.014) and lower VO_2peak_ than men (27 ± 4 mL O_2_/kg/min for women vs. 34 ± 5 mL O_2_/kg/min for men; *p* < 0.001) (Table 1).

### 3.2. Participants Cardiac Structure and Function Parameters

Regarding cardiac structure, women had smaller LV posterior wall thickness than men (0.78 ± 0.12 vs. 0.88 ± 0.13 cm; *p* < 0.001), interventricular wall thickness (0.81 ± 0.13 vs. 0.89 ± 0.12 cm; *p =* 0.001), LV end diastolic diameter (4.63 ± 0.43 vs. 5.0 ± 0.47 cm; *p* < 0.001), and indexed LV mass (65 ± 13 vs. 73 ± 15 g/m^2^; *p =* 0.003). Regarding cardiac function, women had a higher E/e’ ratio (8.1 ± 1.8 vs. 7.3 ± 2.0; *p =* 0.021) and LV GLS (19.8 ± 0.2.2% vs. 18.6 ± 2.4%; *p =* 0.009) (Table 3).

### 3.3. Cardiometabolic Characteristics in Association with Hypertensive Response to Exercise in Women

Among the 118 women, 28 (24%) developed HRE during maximal cardiorespiratory exercise testing. There was no significant difference in terms of age and parameters of metabolic health between the HRE and NHRE groups. Waist circumference was similar in both groups (101.8 ± 11.2 cm for HRE vs. 98.8 ± 9.2 cm for NHRE; *p =* 0.123). Waist-to-hip ratio was higher in the HRE group (0.93 ± 0.06 vs. 0.90 ± 0.05; *p =* 0.006). There were more postmenopausal women in the HRE group (86% vs. 62%; *p =* 0.02). Regarding cardiorespiratory fitness, there were no differences in the relative VO_2peak_ in both groups (26 ± 4 mL O_2_/kg/min for HRE vs. 28 ± 4 mL O_2_/kg/min for NHRE; *p* > 0.05). Women with HRE had higher resting SBP (120 ± 8 mm Hg vs. 112 ± 9 mm Hg), peak exercise SBP (220 ± 13 vs. 182 ± 19 mm Hg), delta SBP response to exercise (101 ± 14 vs. 70 ± 18 mm Hg), and workload-indexed BP response during exercise (17 ± 4 vs. 11 ± 3 mm Hg/METs) than women without HRE (*p* < 0.001 for all) (Table 4 and Table 5).

### 3.4. Cardiac Structure and Function in Association with Hypertensive Response to Exercise in Women

**Figure 1:**
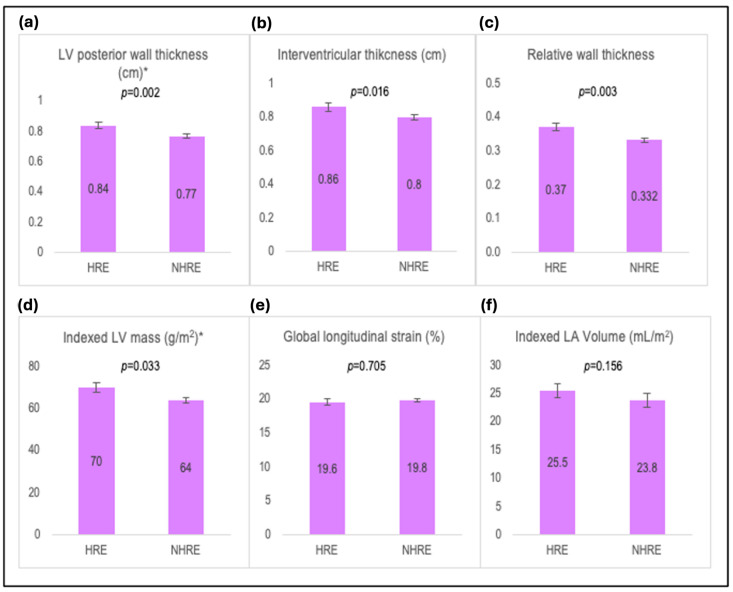
Bar graphs showing comparison of cardiac structure and function between women with and without hypertensive response to exercise. Comparison of (**a**) left ventricular posterior wall thickness, (**b**) interventricular wall thickness, (**c**) relative wall thickness, (**d**) indexed left ventricular mass, (**e**) global longitudinal strain, (**f**) indexed left atrial volume, according to the presence or absence of hypertensive response to exercise among women. Abbreviations: IV, interventricular; LA, left atrial, LV, left ventricular. * *p* < 0.05.

Women in the HRE group had larger LV posterior wall thickness (0.84 ± 0.11 vs. 0.77 ± 0.12 cm), interventricular wall thickness (0.86 ± 0.12 vs. 0.80 ± 0.13 cm), relative wall thickness (0.37 ± 0.06 vs. 0.33 ± 0.06), and indexed LV mass (70 ± 11 vs. 64 ± 13 g/m^2^) compared to women in the NHRE group (*p* < 0.05 for all). Systolic and diastolic function did not differ significantly between the HRE and NHRE groups (*p* > 0.05 for all) (Figure 1, Table 6). There was a numerical but not statistically significant difference in the prevalence of LV concentric remodeling between women with and without HRE (18% vs. 8%; *p =* 0.12).

### 3.5. Cardiometabolic Characteristics in Association with Hypertensive Response to Exercise in Men

Among the 40 men, 9 (23%) developed HRE during exercise testing. There was no significant difference in age, resting SBP, anthropometric measurements, and metabolic health values between men with and without HRE (*p* > 0.05 for all). During cardiorespiratory exercise testing, the HRE group had higher maximal exercise SBP (233 ± 13 vs. 193 ± 20 mm Hg), workload-indexed BP response (13 ± 2 vs. 10 ± 3 mm Hg/MET), and delta SBP response to exercise (108 ± 14 vs. 73 ± 19 mm Hg) compared to the NHRE group (*p* < 0.05 for all). There were no significant differences in relative VO_2peak_ and number of METs between groups (*p* > 0.05 for both) (Table 7 and Table 8).

### 3.6. Cardiac Structure and Function in Association with Hypertensive Response to Exercise in Men

**Figure 2:**
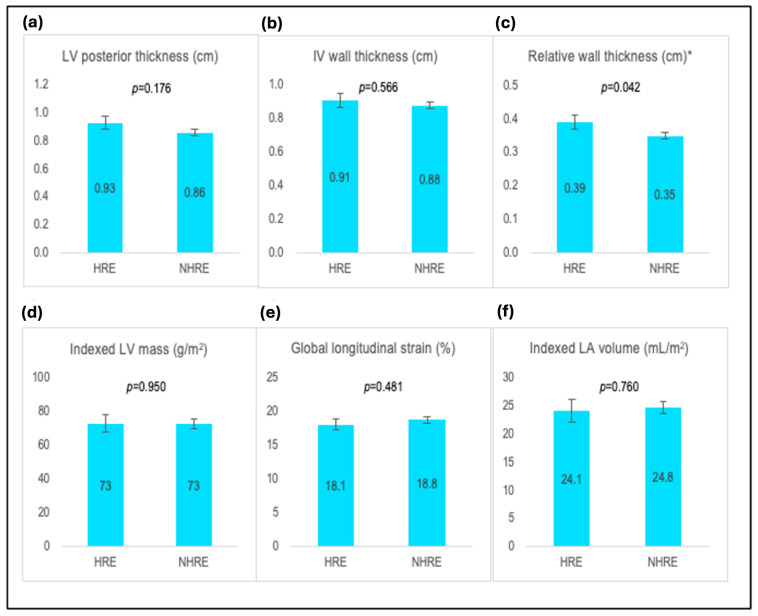
Bar graphs showing comparison of cardiac structure and function between men with and without hypertensive response to exercise. Comparison of (**a**) left ventricular posterior wall thickness, (**b**) interventricular wall thickness, (**c**) relative wall thickness, (**d**) indexed left ventricular mass, (**e**) global longitudinal strain, and (**f**) indexed left atrial volume according to the presence or absence of hypertensive response to exercise among men. Abbreviations: IV, interventricular; LA, left atrial; LV, left ventricular. * *p* < 0.05.

The HRE group showed larger relative wall thickness (0.39 ± 0.06 vs. 0.35 ± 0.06; *p =* 0.042). No significant differences were found between the HRE and NHRE groups regarding other cardiac structural and functional variables (*p* > 0.05 for all, Figure 2, Table 9).

### 3.7. Association Between Maximal Systolic Blood Pressure and Cardiac Structure and Function

The unadjusted distribution of echocardiographic variables according to maximal SBP per sex group is presented in the Supplemental Figure S1. When accounting for age and BMI, maximal SBP significantly correlated with cardiac remodeling, including LV posterior wall thickness (r = 0.29; *p =* 0.002), interventricular wall thickness (r = 0.23; *p =* 0.016), relative wall thickness (r = 0.26; *p =* 0.006), and indexed LV mass (r = 0.21; *p =* 0.031) in women. After further adjustment for resting SBP and VO_2peak_ in multivariable linear regression analysis, maximal SBP remained significantly associated with LV posterior wall thickness and relative wall thickness. (Table 10) No additional significant associations between maximal SBP and LV systolic or diastolic function were found in the fully adjusted models (*p* > 0.05 for all). In this multivariable model, the variation inflation factor for maximal SBP was 1.75 and maximal variance inflation factor was 2.40 for age.

In men, after adjustment for age and BMI, maximal SBP correlated significantly with relative wall thickness (r = 0.38; *p =* 0.019). In the multivariable linear regression model further adjusted for resting SBP and VO_2peak_, maximal SBP remained significantly associated with relative wall thickness and showed an association with the E/A ratio. (Table 10) No other measurements of cardiac remodeling or function remained significantly associated with maximal SBP in the fully adjusted linear regression models (all *p* > 0.05). In this multivariable model, the variance inflation factor for maximal SBP was the maximal value for the model and was calculated at 1.59. Interaction analyses assessing the modulating effect of sex on the association between echocardiographic variables and maximal SBP were significant regarding the E/A ratio (*p =* 0.03).

## 4. Discussion

This is the first study to evaluate the association between HRE and cardiac remodeling and function, with results specific to sex among individuals with abdominal obesity. Our results suggest that, in the context of abdominal obesity, HRE is associated with subclinical adverse cardiac remodeling, even in the presence of normal resting BP. Considering the high prevalence of HRE in our cohort, these results identify a subgroup of individuals who could be in the very early stages of cardiac disease and potentially be at higher risk of future cardiac disease, including heart failure with preserved LV ejection fraction.

### 4.1. Hypertensive Response to Exercise and Adverse Cardiac Remodeling

Our results are compatible with previous studies that have reported increased indexed LV mass and relative wall thickness in individuals with HRE and normal resting BP [3,28]. Although most values of cardiac structure in our cohort were within normal range, the difference observed between individuals with and without HRE could suggest an early stage of adverse cardiac remodeling in those with HRE. These findings were significant despite comparable clinical characteristics and cardiorespiratory fitness between groups. It has been previously shown that a high proportion of individuals with HRE can also experience masked HTN (normal clinical BP, but elevated ambulatory BP) [5,45]. In our study, we considered maximal SBP obtained during the maximal cardiorespiratory exercise testing for classification of HRE. However, we did not identify the exercise load at which HRE threshold was reached. It is possible that certain individuals developed HRE earlier during their exercise performance, at a submaximal intensity level, which could be related to frequently elevated BP during daily life activities [27,46]. Moreover, participants with HRE had higher resting and pretest BP, as well as higher SBP response per METs during exercise, suggesting that HRE may reflect more extensive abnormalities in vasomotor reactivity and vascular stiffness of the arterial system. Consequently, the frequent pressure overload during exercise may contribute to the development of LV concentric remodeling and to a greater cardiac structural impact [23,24,25]. In the long term, adverse cardiac remodeling in the context of HTN can progress towards LV concentric hypertrophy and heart failure [23,24,25]. An increased LV mass is associated with cardiac diastolic dysfunction and major adverse cardiovascular events [22,47,48].

Few studies have evaluated the association of HRE with markers of cardiac subclinical and clinical function. While we did not observe significant differences in cardiac function between HRE and NHRE in our population, Takamura et al. have previously demonstrated an association of HRE with worse diastolic function [26]. Nevertheless, by evaluating HRE systematically after 6 min of a Bruce protocol, compared to maximal SBP during a maximal effort, it can be surmised that their participants had more aggressive HRE compared to our study sample. Furthermore, their population was older than ours, reflecting participants who may have suffered from HRE for a longer duration and experienced more important consequences on their LV. Conversely, and in alignment with our findings, a study by Mottram et al. did not show significant differences in diastolic function between normotensive individuals with and without HRE [49], although with slightly worse subclinical systolic cardiac function (GLS) in participants with HRE, a finding that was not reproduced in our analysis. It is possible that HRE was less severe in our population, given that we used an age- and sex-specific cut-off as opposed to a non-age-adjusted sex-specific higher cut-off value.

### 4.2. Sex-Specific Association of Hypertensive Response to Exercise and the Left Ventricle

Due to the limited sample size and reduced prevalence of men, the statistical power was limited to assessing sex differences in the association between HRE and cardiac remodeling and function. It is well known that the myocardial response to pressure overload differs between men and women, underscoring the importance of sex-specific reporting of results [50]. For instance, women are more predisposed to LV concentric remodeling than men [51]. In the context of pressure overload, women tend to have smaller cavities and larger wall thickness than men [51], while in the presence of obesity, women tend to have a higher prevalence of LV hypertrophy than men [52]. Furthermore, women exhibit lower resting and exercise BP compared to men, supporting the use of sex-specific diagnostic thresholds to identify HRE [8]. It is noteworthy that BP response to exercise is more closely related to arterial stiffness in women, an independent cardiovascular risk factor as well as contributor to adverse cardiac remodeling [53]. Compatible with large epidemiological studies, women in our cohort had smaller LV dimensions, smaller indexed LV mass, and better LV GLS compared to men. While comparison between men and women could not be performed in our exploratory analysis, we observed a higher number of significant LV structural differences between HRE and NHRE in women, in line with previous observations of a worse LV concentric remodeling response to pressure overload in this subgroup compared to men.

### 4.3. Abdominal Obesity and Hypertensive Response to Exercise

The pathophysiology of HRE is multifactorial and includes higher arterial stiffness, vascular peripheral resistance, worse endothelial dysfunction, increased activation of the sympathetic nervous and renin-angiotensin-aldosterone systems, and enhanced chronic inflammation [4,54,55,56,57,58,59,60,61,62,63]. Excess visceral adipose tissue is an important contributor to these mechanisms while also being associated with HRE [7,18,19,20,21]. Although both HRE and obesity have been associated with adverse LV remodeling, it is noteworthy that our results showed worse markers of remodeling in participants with HRE independently from BMI, suggesting a potentially higher cardiovascular risk in this study group. The rapidly increasing prevalence of obesity in the general population [64] and its impactful burden on future cardiovascular health and mortality bring this condition at the center of new management strategies in cardiovascular prevention [65,66]. It is well established that ectopic adipose tissue deposition, such as visceral fat, contributes significantly to cardiovascular risk [67]. Yet anthropometric measures of abdominal obesity provide an imperfect estimate of visceral adipose tissue deposition and of its metabolic activity [68]. The additional assessment of BP response to exercise could represent a useful, cheap, and non-invasive tool to identify individuals with worse cardiometabolic risk profiles beyond their anthropometric status. Therefore, given the limited availability of healthcare resources and the fact that abdominal obesity is a treatable, reversible condition, the detection of HRE in this population could facilitate the early identification and implementation of preventive strategies to lower future cardiovascular risk among those who would potentially benefit the most [69].

### 4.4. Practical Implications

The results of this exploratory analysis demonstrate a statistically significant association of HRE with adverse cardiac remodeling, albeit with a small size effect. Yet these associations were found among asymptomatic individuals at increased metabolic risk but otherwise considered to be healthy. The preclinical finding of more adverse cardiac remodeling in those with HRE in the context of the hypertriglyceridemic waist phenotype could represent early signs of overwhelmed vasomotor adaptation mechanisms leading to increased risk of hypertension, heart failure, and cardiovascular events. More studies are needed to compare sex differences in cardiac remodeling secondary to HRE, including in the context of the hypertriglyceridemic waist phenotype, and to establish its link with future cardiovascular prognosis. Nonetheless, the assessment of BP response to exercise could represent a realistically implantable strategy to focus preventive efforts in those at higher cardiovascular risk.

### 4.5. Strengths and Limitations

The principal strength of our study lies in the comprehensive evaluation of metabolic health, cardiorespiratory fitness and LV cardiac structure and function, which included waist and hip circumference measurement as well as quantification of the LV subclinical systolic function with GLS. This detailed assessment enabled an in-depth analysis of the potential role of HRE on cardiac health within the context of abdominal obesity. In addition, we used HRE cut-off values that were specific to both age and sex, thus allowing for consideration of how age modulates the BP response to exercise with enhanced precision.

On the other hand, this cross-sectional observational analysis precluded the inference of a causal link between HRE and adverse cardiac remodeling. This study was a non-prespecified secondary analysis of participants’ baseline data who had been enrolled in a randomized control trial. The sample size was not calculated in view of the current hypothesis, and the significance and strength of certain associations may therefore have been underestimated. Moreover, the sample size of the male subgroup was relatively small, which may also have led to underestimation of the association between HRE and LV structure while limiting the possibility of interaction testing between sex and HRE. Also, because abdominal obesity was an inclusion criterion in the primary analysis of this study, the effect of the interaction of HRE and abdominal obesity on echocardiographic measurements could not be analyzed. Larger prospective imaging studies in more diverse populations are needed to better understand the potential impacts of HRE on cardiac structure and function specific to each sex. The BP measurement protocol was not initially established to obtain the exact maximal BP throughout the exercise test. When participants could not complete a full stage, the maximal SBP value was the one taken from the previous stage or early in recovery. Hence, it is possible that the maximal BP was underestimated in these cases.

## 5. Conclusions

Individuals with HRE, a resting BP < 140/90 mm Hg, and abdominal obesity display findings of more adverse cardiac remodeling. These characteristics combined could potentially identify high-risk individuals in the early stages of cardiac disease and at higher risk of future heart failure and cardiovascular events. Longitudinal imaging studies are needed to elucidate the extent of cardiovascular consequences resulting from HRE and the potential complimentary role of abdominal obesity along with HRE on future cardiovascular risk.

## Figures and Tables

**Table 1:** Anthropometric and metabolic characteristics in women and men with abdominal obesity.

	Women (*n =* 118)	Men (*n =* 40)	All Participants (*n =* 158)	*p*-Value *
Age—years	54 ± 11	51 ± 11	53 ± 11	0.14
BMI—kg/m^2^	30.1 ± 4.4	30.9 ± 2.6	30.2 ± 4.0	0.29
Waist circumference—cm	99.5 ± 9.8	107.9 ± 7.2	101.6 ± 9.9	<0.001
Waist-to-hip ratio	0.91 ± 0.06	1.01 ± 0.05	0.93 ± 0.07	<0.001
Resting SBP—mm Hg	114 ± 9	122 ± 8	116 ± 10	<0.001
Resting DBP—mm Hg	72 ± 7	75 ± 8	72 ± 7	0.01
Resting HR—BPM	69 ± 8	67 ± 10	69 ± 9	0.27
Metabolic Variables				
Fasting glucose—mmol/L	5.3 ± 0.6	5.5 ± 0.5	5.3 ± 0.6	0.06
HbA1c—%	5.3 ± 0.4	5.3 ± 0.2	5.3 ± 0.3	0.77
HOMA-IR—index	2.7 ± 1.4	3.6 ± 1.9	2.9 ± 1.6	0.005
TG—mmol/L	2.0 ± 0.8	2.2 ± 0.9	2.1 ± 0.8	0.17
HDL-C—mmol/L	1.4 ± 0.3	1.0 ± 0.2	1.3 ± 0.4	<0.001
LDL-C—mmol/L	3.4 ± 0.8	3.3 ± 1.0	3.4 ± 0.8	0.33
Apo-B—g/L	1.1 ± 0.2	1.1 ± 0.2	1.1 ± 0.2	0.62
Hs-CRP—mg/L	3.7 ± 3.5	2.5 ± 2.3	3.4 ± 3.3	0.045

Data are presented as mean ± standard deviation. Abbreviations: Apo-B, apolipoprotein B; BMI, body mass index; HbA1c, glycated hemoglobin; HDL-C, high-density lipoprotein cholesterol; HOMA-IR, homeostasis model assessment of insulin resistance; LDL-C, low-density lipoprotein cholesterol; Hs-CRP, high-sensitivity C-reactive protein; TG, triglycerides. * *p*-value for independent sample Student’s *t* test between women and men.

**Table 2:** Cardiopulmonary exercise testing results in women and men with abdominal obesity.

	Women (*n =* 118)	Men (*n =* 40)	All Participants (*n =* 158)	*p*-Value *
Cardiorespiratory Exercise Testing				
Pretest SBP—mm Hg	135 ± 15	137 ± 13	136 ± 15	0.420
Pretest DBP—mm Hg	82 ± 7	82 ± 9	82 ± 8	0.980
Pretest HR—BPM	77 ± 11	76 ± 15	77 ± 12	0.742
Maximal SBP—mm Hg	191 ± 24	202 ± 25	194 ± 25	0.014
Maximal DBP—mm Hg	77 ± 8	76 ± 10	77 ± 9	0.496
Maximal HR—BPM	170 ± 12	172 ± 14	170 ± 13	0.362
Maximal double product —BPM * mm Hg	32,389 ± 4149	34,742 ± 4929	32,985 ± 4463	0.004
Maximal METs—METs	7.8 ± 1.2	9.7 ± 1.3	8.3 ± 1.5	<0.001
Relative VO_2peak_— mL O_2_/kg/min	27 ± 4	34 ± 5	29 ± 5	<0.001
Workload-indexed BP response—mm Hg/METs	13 ± 4	10 ± 3	12 ± 4	0.001
Delta SBP—mm Hg	77 ± 21	81 ± 23	78 ± 22	0.405

Data are presented as mean ± standard deviation. Abbreviations: DBP, diastolic blood pressure; Delta: difference between maximal SBP and resting SBP; HR, heart rate; SBP, systolic blood pressure. * *p*-value for independent sample Student’s *t* test between women and men.

**Table 3:** Cardiac structure and function in women and men with abdominal obesity.

	Women (*n =* 118)	Men (*n =* 40)	All Participants (*n =* 158)	*p*-Value *
Cardiac structure				
LV posterior wall thickness—cm	0.78 ± 0.12	0.88 ± 0.13	0.81 ± 0.13	<0.001
Interventricular wall thickness—cm	0.81 ± 0.13	0.89 ± 0.12	0.83 ± 0.13	0.001
LV end-diastolic diameter—cm	4.63 ± 0.43	5.0 ± 0.47	4.73 ± 0.46	<0.001
Relative wall thickness	0.34 ± 0.06	0.35 ± 0.06	0.34 ± 0.06	0.23
Indexed LV mass—g/m^2^	65 ± 13	73 ± 15	67 ± 14	0.003
Concentric remodeling, n (%)	12 (10%)	6 (15%)	18 (11%)	0.29
Cardiac function				
E/A ratio	1.1 ± 0.3	1.2 ± 0.3	1.1 ± 0.3	0.449
E/e’ ratio	8.1 ± 1.8	7.3 ± 2.0	7.9 ± 1.9	0.021
Lateral e’ velocity—cm/s	10.8 ± 3.0	11.2 ± 2.5	10.9 ± 2.9	0.396
Septal e’ velocity—cm/s	8.2 ± 2.1	8.2 ± 1.6	8.2 ± 2.0	0.88
Indexed left atrial volume—mL/m^2^	24.2 ± 5.5	24.6 ± 5.8	24.3 ± 5.6	0.683
LV GLS—%	19.8 ± 2.2	18.6 ± 2.4	19.5 ± 2.3	0.009
LV ejection fraction—%	64 ± 4	63 ± 3	64 ± 4	0.111

Data are presented as mean ± SD. Abbreviations: GLS, global longitudinal strain; LV, left ventricle. * *p*-value for independent sample Student’s *t* test between women and men.

**Table 4:** Anthropometric and metabolic characteristics in women with and without hypertensive response to exercise.

	Women (*n =* 118)	Women HRE (*n =* 28)	Women NHRE (*n =* 90)	*p*-Value *
Age—years	54 ± 11	57 ± 10	53 ± 11	0.12
BMI—kg/m^2^	30.1 ± 4.4	30.5 ± 4.3	29.9 ± 4.5	0.63
Waist circumference—cm	99.5 ± 9.8	101.8 ± 11.2	98.8 ± 9.2	0.12
Waist-to-hip ratio	0.91 ± 0.06	0.93 ± 0.06	0.90 ± 0.05	0.006
Resting SBP—mm Hg	114 ± 9	120 ± 8	112 ± 9	<0.001
Resting DBP—mm Hg	72 ± 7	72 ± 6	72 ± 7	0.52
Resting HR—BPM	69 ± 8	67 ± 7	70 ± 8	0.326
Metabolic Variables				
Fasting glucose—mmol/L	5.3 ± 0.6	5.4 ± 0.7	5.24 ± 0.51	0.26
HbA1c—%	5.3 ± 0.4	5.4 ± 0.4	5.3 ± 0.4	0.53
HOMA-IR—Index	2.7 ± 1.4	2.8 ± 1.8	2.7 ± 1.3	0.75
TG—mmol/L	2.0 ± 0.8	2.1 ± 0.8	2.0 ± 0.7	0.92
HDL-C—mmol/L	1.4 ± 0.3	1.3 ± 0.3	1.4 ± 0.4	0.61
LDL-C—mmol/L	3.4 ± 0.8	3.7 ± 0.6	3.3 ± 0.8	0.16
Apo-B—g/L	1.1 ± 0.2	1.2 ± 0.1	1.1 ± 0.2	0.25
Hs-CRP—mg/L	3.7 ± 3.5	3.4 ± 2.3	3.8 ± 3.8	0.62

Data are presented as mean ± SD. Abbreviations as in Table 1. * *p*-value for independent sample Student’s *t* test between women HRE and women NHRE.

**Table 5:** Cardiorespiratory exercise testing results in women with and without hypertensive response to exercise.

	Women (*n =* 118)	Women HRE (*n =* 28)	Women NHRE (*n =* 90)	*p-*Value *
Cardiorespiratory Exercise Testing				
Pretest SBP—mm Hg	135 ± 15	145 ± 11	132 ± 15	<0.001
Pretest DBP—mm Hg	82 ± 7	84 ± 9	82 ± 7	0.13
Pretest HR—BPM	77 ± 11	74 ± 11	78 ± 11	0.15
Maximal SBP—mm Hg	191 ± 24	220 ± 13	182 ± 19	<0.001
Maximal DBP—mm Hg	77 ± 8	79 ± 10	77 ± 8	0.14
Maximal HR—BPM	170 ± 12	167 ± 15	171 ± 11	0.18
Maximal double product —BPM*mm Hg	32,389 ± 4149	36,770 ± 3154	31,027 ± 3420	<0.001
Maximal METs—n	7.8 ± 1.2	7.4 ± 1.2	7.9 ± 1.2	0.09
Relative VO_2peak_— mL O_2_/kg/min	27 ± 4	26 ± 4	28 ± 4	0.09
Workload-indexed BP response—mm Hg/METs	13 ± 4	17 ± 4	11 ± 3	<0.001
Delta SBP—mm Hg	77 ± 21	101 ± 14	70 ± 18	<0.001

Data are presented as mean ± SD. Abbreviations as in Table 2. * *p*-value for independent sample Student’s *t* test between women HRE and women NHRE.

**Table 6:** Comparison of cardiac structure and function in women with and without hypertensive response to exercise.

	Women (*n =* 118)	Women HRE (*n =* 28)	Women NHRE (*n =* 90)	*p-*Value *
Cardiac structure				
LV posterior wall thickness—cm	0.78 ± 0.12	0.84 ± 0.11	0.77 ± 0.12	0.002
Interventricular wall thickness—cm	0.81 ± 0.13	0.86 ± 0.12	0.80 ± 0.13	0.016
LV diameter—cm	4.63 ± 0.43	4.60 ± 0.40	4.64 ± 0.43	0.66
Relative wall thickness	0.34 ± 0.06	0.37 ± 0.06	0.33 ± 0.06	0.003
Indexed LV mass—g/m^2^	65 ± 13	70 ± 11	64 ± 13	0.03
Concentric remodeling, *n* (%)	12 (10%)	5 (17.9)	7 (7.8)	0.12
Cardiac function				
E/A ratio	1.1 ± 0.3	1.0 ± 0.3	1.2 ± 0.3	0.14
E/e’ ratio	8.1 ± 1.8	8.9 ± 2.0	7.9 ± 1.8	0.054
Lateral e’ velocity—cm/s	10.8 ± 3.0	10.0± 2.9	11.0 ± 3.0	0.14
Septal e’ velocity—cm/s	8.2 ± 2.1	7.5 ± 1.8	8.4 ± 2.1	0.06
Indexed left atrial volume—mL/m^2^	24.2 ± 5.5	25.5 ± 4.8	23.8 ± 5.7	0.16
LV GLS—%	19.8 ± 2.2	19.6 ± 2.4	19.8 ± 2.2	0.71
LV ejection fraction—%	64 ± 4	64 ± 3	64 ± 4	0.92

Data are presented as mean ± standard deviation. Abbreviations as in Table 3. * *p*-value for independent sample Student’s *t* test between women HRE and women NHRE.

**Table 7:** Anthropometric and metabolic characteristics in men with and without hypertensive response to exercise.

	Men (*n =* 40)	Men HRE (*n =* 9)	Men NHRE (*n =* 31)	*p*-Value *
Age—years	51 ± 11	52 ± 8	51 ± 12	0.74
BMI—kg/m^2^	30.9 ± 2.6	31.7 ± 2.4	30.6 ± 2.7	0.32
Waist circumference—cm	107.9 ± 7.2	108.7 ± 9.9	107.7 ± 6.4	0.70
Waist-to-hip ratio	1.01 ± 0.05	1.01 ± 0.03	1.01 ± 0.05	0.90
Resting SBP—mm Hg	122 ± 8	125 ± 7	121 ± 9	0.20
Resting DBP—mm Hg	75 ± 8	76 ± 6	75 ± 8	0.68
Resting HR—BPM	67 ± 10	67 ± 13	67 ± 10	0.99
Metabolic Variables				
Fasting glucose—mmol/L	5.5 ± 0.5	5.7 ± 0.6	5.4 ± 0.5	0.17
HbA1c—%	5.3 ± 0.2	5.4 ± 0.3	5.3 ± 0.2	0.61
HOMA-IR—Index	3.6 ± 1.9	4.6 ± 2.8	3.3 ± 1.6	0.06
TG—mmol/L	2.2 ± 0.9	2.6 ± 1.1	2.1 ± 0.8	0.17
HDL-C—mmol/L	1.0 ± 0.2	1.0 ± 0.2	1.1 ± 0.3	0.22
LDL-C—mmol/L	3.3 ± 1.0	3.4 ± 1.0	3.2 ± 1.0	0.70
Apo-B—g/L	1.1 ± 0.2	1.2 ± 0.3	1.1 ± 0.2	0.48
Hs-CRP—mg/L	2.5 ± 2.3	1.9 ± 1.4	2.7 ± 2.5	0.37

Data are presented as mean ± standard deviation. Abbreviations as in Table 1. * *p*-value for independent sample Student’s *t* test between men HRE and men NHRE.

**Table 8:** Cardiorespiratory exercise testing results in men with and without hypertensive response to exercise.

	Men (*n =* 40)	Men HRE (*n =* 9)	Men NHRE (*n =* 31)	*p*-Value *
Cardiorespiratory Exercise Testing				
Pretest SBP—mm Hg	135 ± 15	145 ± 11	132 ± 15	<0.001
Pretest DBP—mm Hg	82 ± 7	84 ± 9	82 ± 7	0.13
Pretest HR—BPM	77 ± 11	74 ± 11	78 ± 11	0.15
Maximal SBP—mm Hg	191 ± 24	220 ± 13	182 ± 19	<0.001
Maximal DBP—mm Hg	77 ± 8	79 ± 10	77 ± 8	0.14
Maximal HR—BPM	170 ± 12	167 ± 15	171 ± 11	0.18
Maximal double product —BPM * mm Hg	32,389 ± 4149	36,770 ± 3154	31,027 ± 3420	<0.001
Maximal METs—*n*	7.8 ± 1.2	7.4 ± 1.2	7.9 ± 1.2	0.09
Relative VO_2peak_—mL O_2_/kg/min	27 ± 4	26 ± 4	28 ± 4	0.09
Workload-indexed BP response—mm Hg/METs	13 ± 4	17 ± 4	11 ± 3	<0.001
Delta SBP—mm Hg	77 ± 21	101 ± 14	70 ± 18	<0.001

Data are presented as mean ± standard deviation. Abbreviations as in Table 2. * *p*-value for independent sample Student’s *t* test between men HRE and men NHRE.

**Table 9:** Comparison of cardiac structure and function in men with and without hypertensive response to exercise.

	Men (*n =* 40)	Men HRE (*n =* 9)	Men NHRE (*n =* 31)	*p*-Value *
Cardiac Structure				
LV posterior wall thickness—cm	0.88 ± 0.13	0.93 ± 0.14	0.86 ± 0.13	0.18
Interventricular wall thickness—cm	0.89 ± 0.12	0.91 ± 0.13	0.88 ± 0.12	0.57
LV diameter—cm	5.00 ± 0.47	4.81 ± 0.54	5.05 ± 0.44	0.183
Relative wall thickness	0.35 ± 0.06	0.39 ± 0.06	0.35 ± 0.06	0.042
Indexed LV mass—g/m^2^	73 ± 15	73 ± 15	73 ± 16	0.95
Concentric remodeling, *n* (%)	6 (15%)	3 (33.3)	3 (7.5)	0.08
Cardiac Function				
E/A ratio	1.2 ± 0.3	1.2 ± 0.2	1.2 ± 0.4	0.89
E/e’ ratio	7.3 ± 2.0	7.3 ± 1.5	7.2 ± 2.1	0.85
Lateral e’ velocity—cm/s	11.2 ± 2.5	11.4 ± 2.2	11.2 ± 2.6	0.81
Septal e’ velocity—cm/s	8.2 ± 1.6	8.2 ± 1.4	8.2 ± 1.6	0.93
Indexed left atrial volume—mL/m^2^	24.6 ± 5.8	24.1 ± 6.0	24.8 ± 5.8	0.76
LV GLS—%	18.6 ± 2.4	18.1 ± 2.5	18.8 ± 2.4	0.48
LV ejection fraction—%	63 ± 3	62 ± 2	63 ± 4	0.41

Data are presented as mean ± standard deviation. Abbreviations as in Table 3. * *p*-value for independent sample Student’s *t* test between men HRE and men NHRE.

**Table 10:** Multivariate linear regression models of the association between maximal systolic blood pressure and echocardiographic variables.

.	Women		Men	
	Adjusted *β* [95% CI] * for Maximal SBP	*p*-Value	Adjusted *β* [95% CI] * for Maximal SBP	*p*-Value
LV posterior wall thickness—cm	0.001 [0.000 to 0.423]	0.01	0.001 [−0.001 to 0.003]	0.37
Interventricular wall thickness—cm	0.001 [0.000 to 0.002]	0.064	0.000 [−0.002 to 0.002]	0.94
LV diameter—cm	0.000 [−0.003 to 0.004]	0.84	−0.008 [−0.015 to −0.001]	0.02
Relative wall thickness	0.001 [0.000 to 0.001]	0.03	0.001 [0.000 to 0.002]	0.03
Indexed LV mass—g/m^2^	0.12 [−0.01 to 0.25]	0.06	−0.13 [−0.37 to 0.11]	0.28
E/A ratio	0.000 [−0.002 to 0.002]	0.94	0.005 [0.000 to 0.009]	0.039
E/e’ ratio	0.009 [−0.007 to 0.025]	0.25	0.002 [−0.031 to 0.035]	0.90
Lateral e’ velocity—cm/s	−0.02 [−0.04 to 0.00]	0.08	0.02 [−0.02 to 0.06]	0.31
Septal e’ velocity—cm/s	−0.01 [−0.03 to 0.00]	0.12	0.02 [−0.01 to 0.04]	0.18
Indexed left atrial volume—mL/m^2^	0.03 [−0.03 to 0.09]	0.30	−0.009 [−0.098 to 0.079]	0.83
LV GLS—%	0.003 [−0.020 to 0.025]	0.82	−0.02 [−0.06 to 0.03]	0.49
LV ejection fraction—%	0.003 [−0.038 to 0.044]	0.88	0.01 [−0.04 to 0.07]	0.59

Abbreviations: CI, confidence interval; GLS, global longitudinal strain; LV, left ventricle; SBP, systolic blood pressure. * Models adjusted for age, body mass index, resting systolic blood pressure, and exercise peak oxygen consumption.

## Data Availability

Denominalized data will be available upon request.

## Supplementary Materials

The following supporting information can be downloaded at: https://www.mdpi.com/article/10.3390/jcm14010016/s1, Figure S1: Scatterplots of the association between maximal systolic blood pressure and echocardiographic variables of left ventricular structure and function with simple linear regression, for women and men with abdominal obesity.
