# Supplementary material for: Hypertensive Response to Exercise in Normotensive Men and Women with Abdominal Obesity: Association with Subclinical Adverse Cardiac Remodeling
Source: J Clin Med. 2024 Dec 24;14(1):16. doi: 10.3390/jcm14010016 (PMC11722559; doi:10.3390/jcm14010016)
Supplement: Supplementary file 1 [file jcm-14-00016-s001.zip › jcm-3353282-supplementary.pdf]

Title: Hypertensive Response to Exercise in Normotensive Men and Women with Abdominal Obesity: Association with Subclinical Adverse Cardiac Remodeling

Amélie Paquin[1-2], MD, MSc, PhD(c)*, Marie-Anne Mathieu[1-2], MS*, Chloé Prémont[1-2], Iris Gigleux[3], DtP, MSc, Anne-Sophie Neyron[3], MSc, Maggie Lê-Brassard[3], MSc, Mickaël Martin[1], MSc, Audrey Auclair[1], PhD, Myriam Pettigrew[1], MSc, Robert Ross[4], PhD, Patrick Couture[2,3], MD, PhD, Jean-Pierre Després[1-2-5], PhD, Paul Poirier[1-6], MD, PhD, Benoît Lamarche[3], PhD, Marie-Eve Piché[1-2], MD, PhD.

*A Paquin and MA Mathieu contributed equally to this work.

[1]Institut Universitaire de Cardiologie et de Pneumologie de Québec - Université Laval, Québec, Canada

[2]Faculty of medicine, Laval University, Québec, QC, Canada

[3]Centre Nutrition, santé et société (NUTRISS), Institut sur la nutrition et les aliments fonctionnels (INAF), Université Laval, Québec, QC, Canada

[4] School of Medicine, Division of Endocrinology and Metabolism, Queen's University, Kingston, ON, Canada

[5]VITAM – Centre de recherche en santé durable, Université, Laval, Québec, QC, Canada

[6]Faculty of pharmacy, Laval University, Québec, QC, Canada

SUPPLEMENTAL MATERIAL

- Supplemental Figure S1 p.2

Supplemental Figure

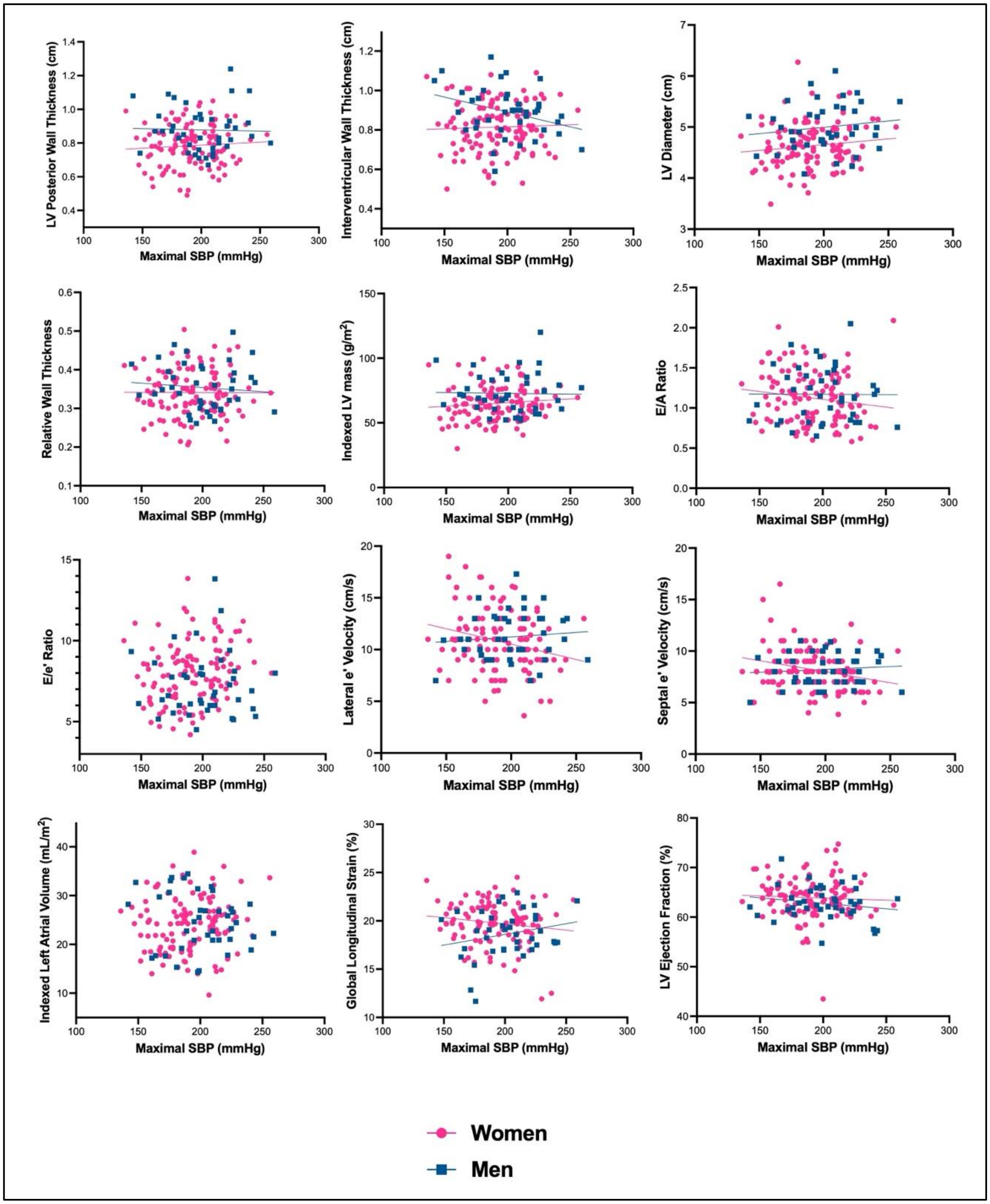

Figure S1. Scatterplots of the association between maximal systolic blood pressure and echocardiographic variables of left ventricular structure and function with simple linear regression, for women and men with abdominal obesity. Abbreviations: LV, left ventricular; SBP, systolic blood pressure.
